# Guided screen for synergistic three-drug combinations

**DOI:** 10.1371/journal.pone.0235929

**Published:** 2020-07-09

**Authors:** Melike Cokol-Cakmak, Selim Cetiner, Nurdan Erdem, Feray Bakan, Murat Cokol

**Affiliations:** 1 Faculty of Engineering and Natural Sciences, Sabanci University, Istanbul, Turkey; 2 Nanotechnology Research and Application Center, Sabanci University, Istanbul, Turkey; Universidade Federal do Rio de Janeiro, BRAZIL

## Abstract

Combinations of three or more drugs are routinely used in various medical fields such as clinical oncology and infectious diseases to prevent resistance or to achieve synergistic therapeutic benefits. The very large number of possible high-order drug combinations presents a formidable challenge for discovering synergistic drug combinations. Here, we establish a guided screen to discover synergistic three-drug combinations. Using traditional checkerboard and recently developed diagonal methods, we experimentally measured all pairwise interactions among eight compounds in *Erwinia amylovora*, the causative agent of fire blight. Showing that synergy measurements of these two methods agree, we predicted synergy/antagonism scores for all possible three-drug combinations by averaging the synergy scores of pairwise interactions. We validated these predictions by experimentally measuring 35 three-drug interactions. Therefore, our guided screen for discovering three-drug synergies is (i) experimental screen of all pairwise interactions using diagonal method, (ii) averaging pairwise scores among components to predict three-drug interaction scores, (iii) experimental testing of top predictions. In our study, this strategy resulted in a five-fold reduction in screen size to find the most synergistic three-drug combinations.

## Introduction

The treatment of many pathogens and tumors involve drug cocktails of three or more combinations. Such combinations are chosen primarily due to their non-overlapping resistance mechanisms [1,2] and the drug interaction (synergy/antagonism) phenotype [3–6]. Resistance is informed primarily by the mechanism of action of individual drugs. However, a drug combination’s interaction phenotype is determined by experimentation and the astronomically large space of possible high-order combinations precludes comprehensive screens [7,8].

Pairwise drug interactions have been traditionally measured by checkerboard assays, where two drugs are combined in a 2D matrix [9,10]. While still the gold standard for discovering drug synergy, this method is both time and resource intensive. A miniaturized checkerboard reduces the experimental cost of the pairwise interaction measurement by reducing the number of tested combinations [11]. However, even with the more efficient checkerboard assays, the testing of high-order interactions is impractical. An alternative way of measuring drug interactions is the diagonal assay, which samples the most informative regions of the checkerboard assay, thereby reducing the resource use [12,13]. Moreover, the diagonal assay can also be used to efficiently measure high-order interactions [14].

Even with the development of efficient interaction assays, a comprehensive screen for all high-order combinations for drugs targeting a given disease remains elusive. Among 10 drugs, there are only 45 pairwise combinations. However, there are 120 three-drug and 252 five-drug combinations. For any set with more than 10 drugs, the experimental screen for all possible high-order combinations quickly becomes unfeasible. However, several recent studies showed that pairwise interactions can provide reliable estimates for high-order interactions [14–17]. Therefore, comprehensive experimental testing of only pairwise interactions, predicting high-order interactions and experimentally validating the most promising predictions may be an efficient method to discover high-order synergies.

When three drugs A, B, and C are combined, the observed (nominal) interaction has four components: Three pairwise interactions (A+B, A+C, B+C) and the interaction resulting specifically from the combination of all three components (A+B+C). This specific interaction resulting only from the high-order combination has been referred to as “emergent interaction [13, 18].” These four components can be expressed as: nominal interaction = emergent interaction + mean (three pairwise interactions). Moreover [13], multiple studies have shown that emergent interactions are rare [6,14]. Therefore, simple mean of pairwise interactions may provide a reliable estimate for nominal three-drug interaction scores.

Here, we tested this approach by first establishing that diagonal assays and checkerboard assays are in agreement for a large set of pairwise interactions among eight compounds. Using the pairwise interaction scores, we predicted three-drug interaction scores, which we experimentally confirmed. Our results suggest that a search for high-order interactions may require only diagonal assays, and checkerboard assays can be used for validation purposes. Moreover, our results show that the strongest high-order synergies are concentrated among the top predictions, which greatly reduces the experimental search space for high-order drug synergies.

We used *Erwinia amylovora*, which causes fire blight, as the biological agent in our study. This bacterial pathogen costs to the agricultural economy more than $100 million in the USA [19] per year by destroying young apple and pear trees. We used a set of eight compounds for the screens described in this study. Six of these compounds (copper sulphate, gentamicin, kasugamycin, oxolinic acid, oxytetracycline, and streptomycin) have been previously used against fire blight [20–22]. Pentamidine, which has been found to be frequently synergistic in past screens, was also included in this list to increase the likelihood of finding synergistic interactions [23, 24]. Previous studies have suggested a link between detergent compounds and increased synergy [25] in foodborne pathogens. Therefore, we included SDS, a detergent, in our interaction screen. Although the current study focuses on *E*. *amylovora* and eight compounds against this pathogen, our guided screen can be used to find synergistic three-drug combinations in other biological and chemical contexts.

## Results

### Screen of all pairwise combinations among 8 compounds using miniaturized checkerboard assays

**Fig 1:**
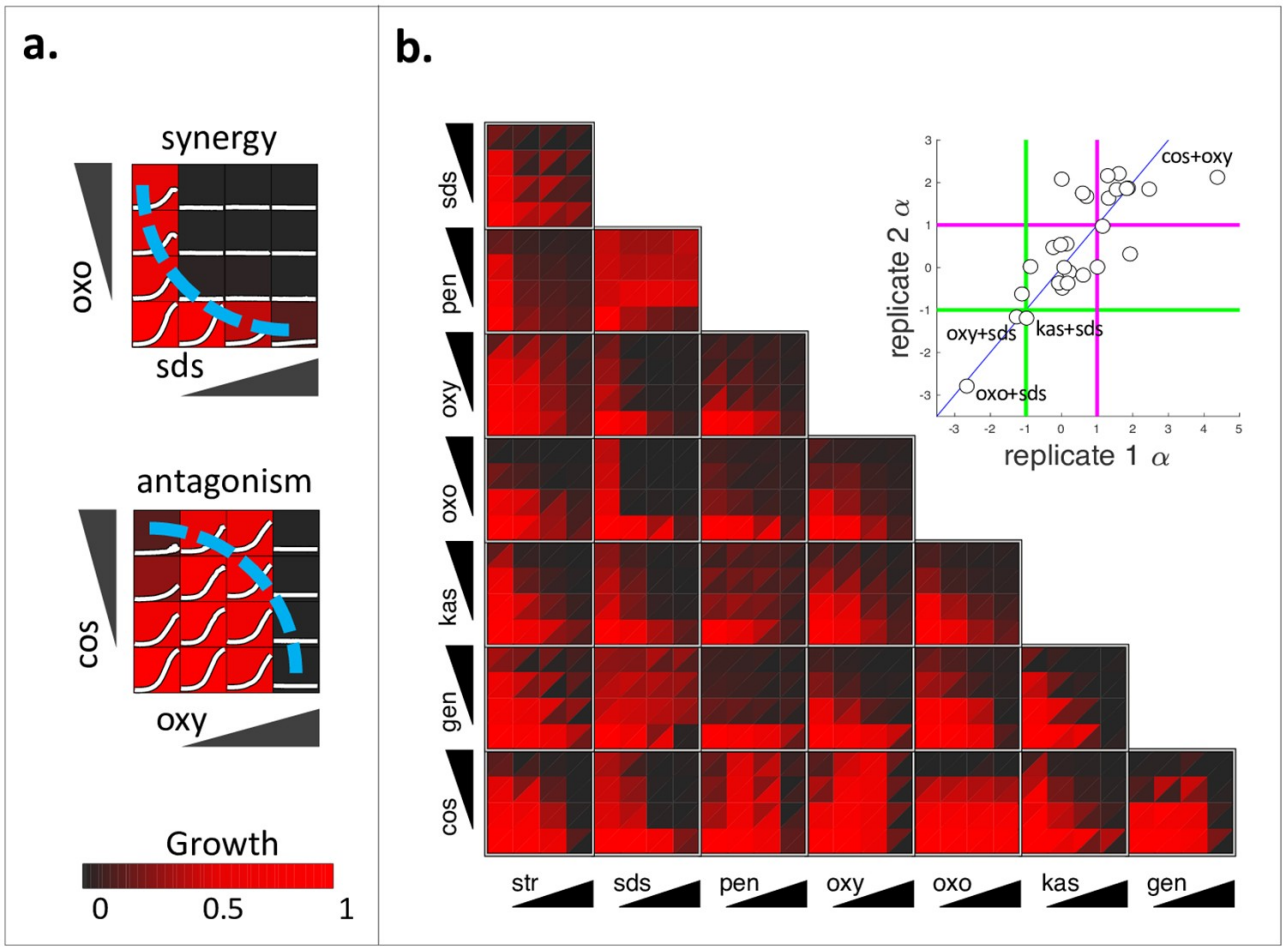
Measurement of all pairwise interactions among eight drugs in *E*. *amylovora*, using checkerboard assays. (a) In these assays, cells are grown in a 4x4 matrix of the combinations of two drugs. The concentration of each drug linearly increases from 0 to the minimum inhibitory concentration (MIC) in one axis. Here, 16-hour growth curves are shown in white for a synergistic pair (oxo+SDS) and an antagonistic pair (cos+oxy) observed in our screen. Growth is defined as the area under the growth curve (AUC) normalized to the AUC of the no drug condition and is represented by a black (no growth) to red (growth in no drug condition) colorbar. We identified the largest isophenotypic contour that connects the observed phenotypes in single drugs. When this isophenotypic contour is linear, two drugs are additive; concave or convex contours (shown in blue) correspond to synergy (oxo+SDS) or antagonism (cos+oxy), respectively. The three-letter abbreviations for all drugs were given in Table 1. (b) Observed growth in the 4x4 checkerboard assays for all 28 pairwise combinations among eight drugs are shown as heatmaps. In each combination, observed growth in two replicates are shown as two triangles. For each checkerboard assay, the concavity of the largest isophenotypic contour was quantified as α, which is negative, 0 or positive for synergistic, additive or antagonistic pairs, respectively. The inset shows a scatter plot for the replicate α scores which showed strong correlation (Spearman r = 0.73, p-value = 1.5 x 10^−5^). Green and magenta lines correspond to synergy (α < -1) and antagonism (α > 1) thresholds. SDS exhibited strong synergy with oxo, oxy and kas. Most combinations were antagonistic, in agreement with previous pairwise interaction screens.

In these assays, *E*. *amylovora* cells were grown in 4x4 grids where the concentration of each drug is linearly increased along each axis from zero to close to Minimum Inhibitory Concentration (MIC). Growth at each concentration combination was normalized to growth at the no drug condition (see Methods [11]). The concavity of isophenotypic growth contours are analyzed to obtain synergy scores [9, 24]. Under the Loewe additivity model, drug combinations with linear isophenotypic contours are defined as additive (non-interacting). When the isophenotypic contour is concave, relatively less drug is needed to achieve the same effect, defined as synergy. Conversely, convex isophenotypic contours correspond to drug antagonism. Names, abbreviation and top concentration for the 8 drugs used in this study are given in Table 1. Example experimental results for a synergistic pair (oxo+SDS) and an antagonistic pair (cos+oxy) are shown in Fig 1a.

**Table 1:** Names, abbreviation, top concentration and class of the drugs used in this study.

COMPOUNDS	ABBREVIATION	MIC (MG/ML)	CLASS
**COPPER (II) SULFATE**	**cos**	**0.5**	**Inorganic compound**
**GENTAMICIN SULFATE**	**gen**	**0.003**	**Aminoglycoside antibiotic**
**KASUGAMYCIN**	**kas**	**0.14**	**Aminoglycoside antibiotic**
**OXOLINIC ACID**	**oxo**	**0.00003**	**Quinolone antibiotic**
**OXYTETRACYCLINE**	**oxy**	**0.0002**	**Tetracycline antibiotic**
**PENTAMIDINE**	**pen**	**0.016**	**Antiprotozoal antibiotic**
**SODIUM DODECYL SULFATE**	**SDS**	**0.15**	**Synthetic organic compound**
**STREPTOMYCIN SULFATE**	**str**	**0.0042**	**Aminoglycoside antibiotic**

We conducted miniaturized checkerboard assays for all 28 pairwise combinations of 8 drugs, in duplicate. Fig 1b shows all the growth measurements for all experiments for both duplicates, which show remarkable agreement. We observe that concave isophenotypic contours (synergy) are rare while convex contours (antagonism) are common, in agreement with numerous studies [8, 23, 24]. The few synergies involve SDS, with pairwise combinations of oxy, kas or oxo showing striking concavity. Cos was antagonistic with all drugs including SDS. We used a previously established score (α) to quantify the concavity of the largest isophenotypic contour observed in each miniaturized checkerboard assay [26]. α is 0 for linear (additive) pairwise combinations; negative or positive for concave (synergistic) or convex (antagonistic) combinations. Fig 1b inset shows the duplicate α scores obtained for all pairs. Two replicate scores significantly correlated (Spearman r = 0.73, p < 0.01), supporting the reliability of the checkerboard screen results.

### Screen of all pairwise combinations among 8 compounds using diagonal assays

**Fig 2:**
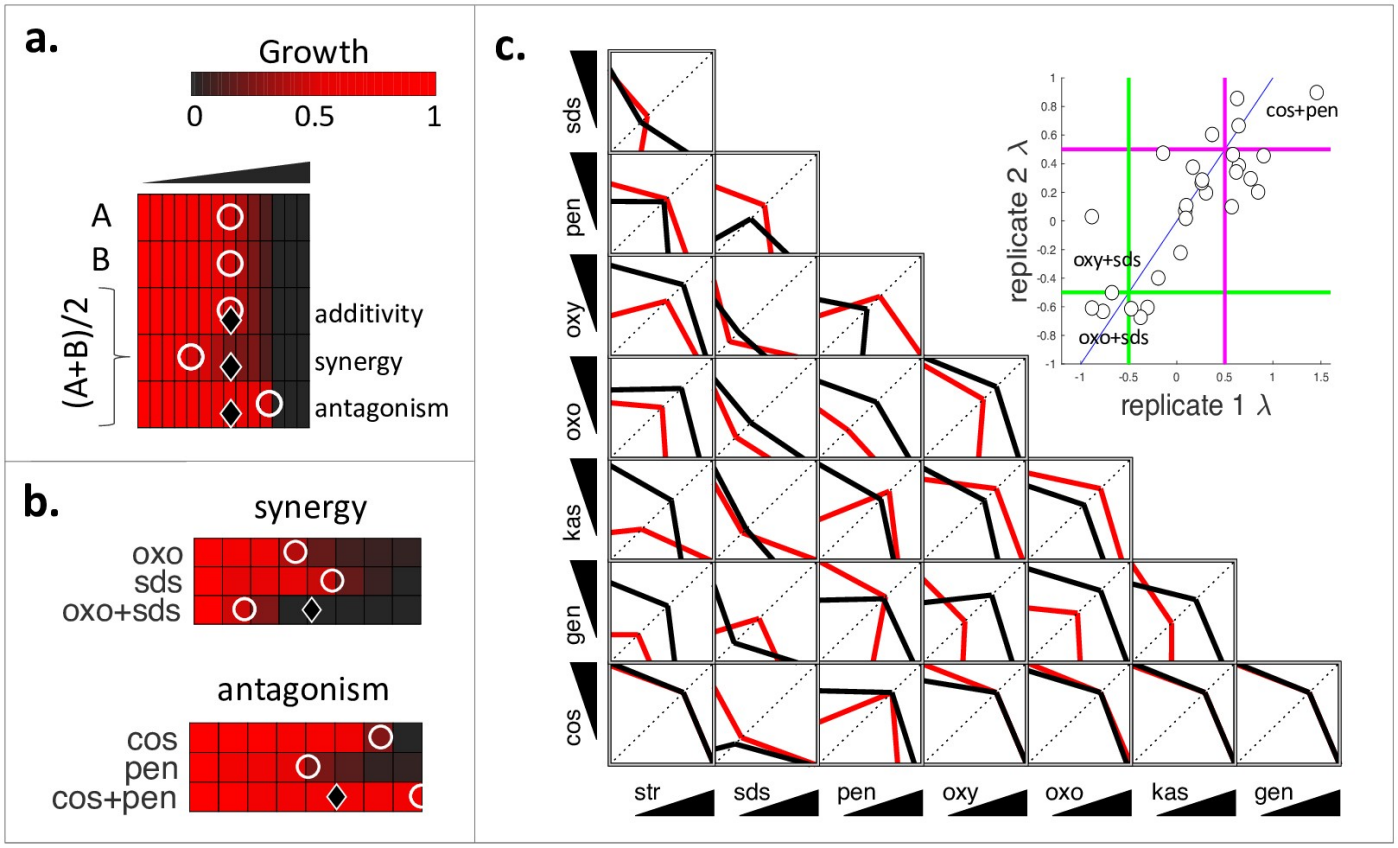
Measurement of all pairwise interactions among eight drugs in *E*. *amylovora* using diagonal assays. (a) In these assays, cells are grown in linearly increasing doses of two single drugs (A and B) and an equipotent mixture of these two drugs. Growth is represented by a black (no growth) to red (growth in no drug condition) colorbar. The observed dose that results in 50% growth inhibition (IC50) is indicated by a white circle. The expected IC50 of the combination is indicated by a black triangle. When drugs are mixed, the observed IC50 of the combination may be lower, equal or higher than the expected IC50, corresponding to synergy, additivity or antagonism, respectively. λ score is defined by log2(obs IC50 / exp IC50), hence negative, zero or positive values correspond to synergy, additivity or antagonism, respectively. (b) Examples of observed synergy and antagonism. The observed IC50 of oxo+SDS combination is smaller than the observed IC50s of single compounds and the expectation calculated for the combination, indicating synergy. cos+pen has almost no effect while both single drugs cos and pen have growth inhibition effects, indicating antagonism. The three-letter abbreviations for all drugs were given in Table 1. (c) Summary of diagonal method results for all pairwise interactions. We projected the observed IC50 in single drugs and two-drug combination in each experiment to a 2D matrix and connected these three points to construct an isophenotypic contour. Red and black contours correspond to two replicates. Similar to the checkerboard assay interpretation, concave or convex contours indicate that the drug combination is synergistic or antagonistic, respectively. The inset shows that replicate λ scores strongly correlate (Spearman r = 0.79, P-value = 2.2 x 10^−6^). Green and magenta lines correspond to synergy (λ < -0.5) and antagonism (λ > 0.5) thresholds.

In these assays, cells are grown in increasing concentrations of individual compounds to determine the dose fractions that result in a given phenotype, such as 50% growth inhibition. Using these values, an expected dose-fraction that would result in the same phenotype for a 1:1 mixture of two compounds is computed under the assumption of additivity among these compounds. To assess synergy, the experimental dose-fraction obtained from a 1:1 mixture of two compounds is compared with the expected dose-fraction. If observed dose-fraction is smaller than, equal to, or larger than the expected dose-fraction, synergy, additivity or antagonism is concluded, respectively (Fig 2a) [12,13]. In Fig 2b, experimental results for a synergistic pair (oxo+SDS) and antagonistic pair (cos+pen) are shown.

We conducted diagonal assays for all 28 pairwise combinations of 8 drugs, in duplicate. Fig 2c shows a visual representation of the data obtained from these assays, where the observed dose-fraction for all single and pairwise combinations are projected on a 2D matrix. This representation allows to approximate the concavity of the isophenotypic contour as determined by the efficient sampling of the diagonal assay. Similar to checkerboard assays, concave or convex contours correspond to synergy or antagonism, respectively. cos was antagonistic in all experiments. In agreement with the checkerboard assays, antagonism was common. We used a previously established score (λ) to quantify the synergy for each experiment, where λ = log_2_(observed dose-fraction / expected dose-fraction). In this setting, a score of 0 corresponds to additivity. Negative and positive scores correspond to synergy or antagonism, respectively. Fig 2c inset shows that the duplicate λ scores for all pairs significantly correlated (Spearman r = 0.79, p < 0.01), supporting the reliability of the diagonal assay results. Next, we compared the α scores obtained from checkerboard assay screen with the λ scores obtained from diagonal assay screen. We used the mean of α or λ values for each pair and found that α and λ scores significantly correlated (Spearman r = 0.53, p < 0.01), supporting the use of diagonal assays as an efficient proxy for checkerboard assays in measuring drug interactions.

### Prediction and testing of three-drug interactions using pairwise interaction scores

**Fig 3:**
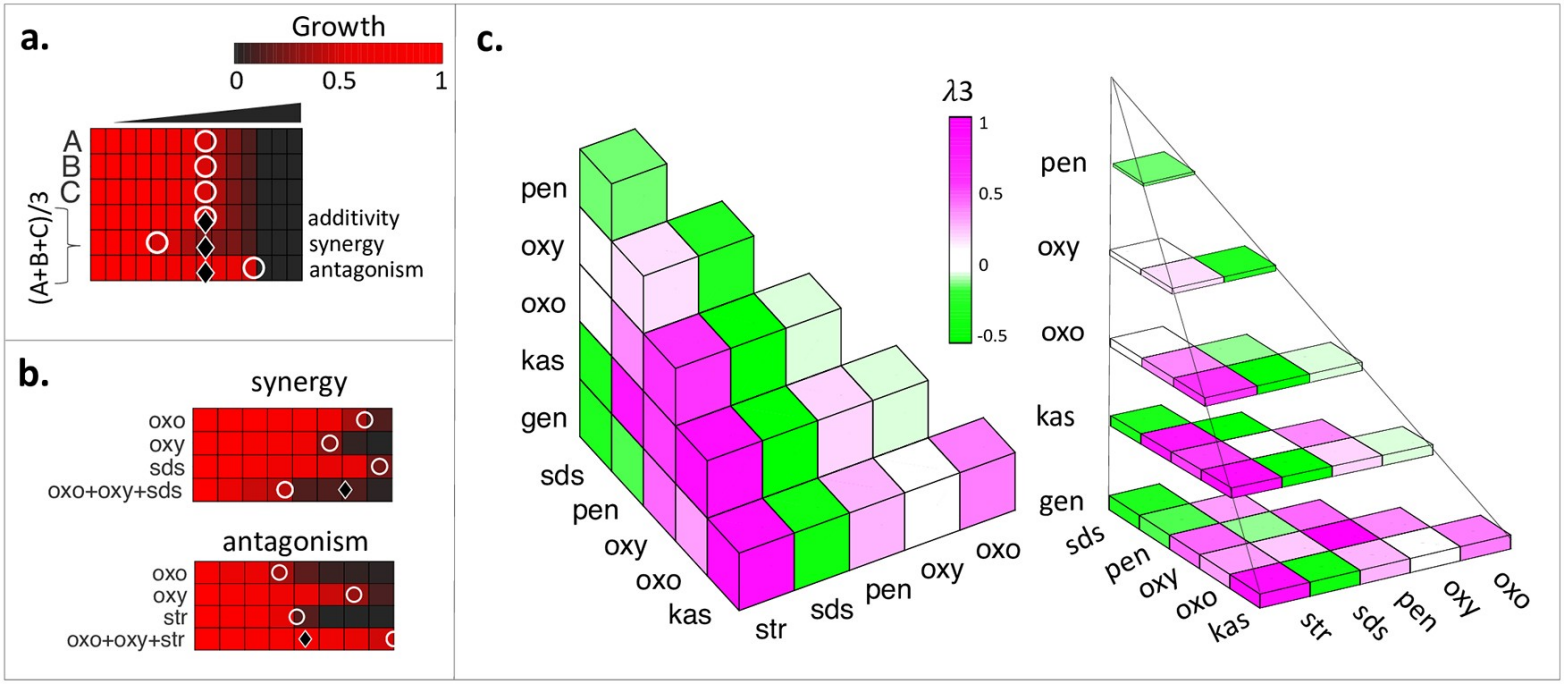
Measurement of all three-drug drug interactions among seven drugs in *E*. *amylovora*, using diagonal assays. (a) Similar to the diagonal assay for pairwise interactions, cells are grown in linearly increasing doses of three single drugs and one equipotent mixture of these three drugs. An expected IC50 is calculated using the IC50 of the individual drugs. If observed IC50 is smaller or larger than expected IC50, synergy or antagonism is concluded, respectively. (b) All three-way interactions are represented as 3D heatmap. Green, white or magenta boxes correspond to synergistic, additive or antagonistic three-drug drug combinations. On the right, the 3D heatmap is shown as layers to assist visualization.

For all three-drug combinations of these 8 antibiotics, we generated predictions by using the arithmetic mean of interaction scores for all 3 pairwise combinations among 3 drugs, as introduced above. For combination A+B+C, the mean (AB, AC, BC) provides the expectation for A+B+C if there is no additional synergy/antagonism associated with the three-drug combination of these drugs. Since we planned to test our predictions using the diagonal method, we only used pairwise λ scores (λ2) for our predictions. The experimental setup and interpretation of three-drug interactions using the diagonal method is similar to the pairwise example with three individual dose responses and 1:1:1 mixture vs. two dose responses and 1:1 mixture (Fig 3a). Fig 3b shows examples for three-drug synergy (oxo+oxy+SDS) and three-drug antagonism (oxo+oxy+str).

To test our predictions, we experimentally tested all three-drug combinations among seven drugs (35 combinations). cos was not included in this screen as all of its interactions were predicted to be antagonistic, hence decreasing the screen size from 56 to 35. The three-drug interaction score (λ3) measured for each combination is shown in Fig 3c. As in the pairwise screen, we found that the most synergistic three-drug combinations involved SDS.

### Comparison of predicted and empirical scores

**Fig 4:**
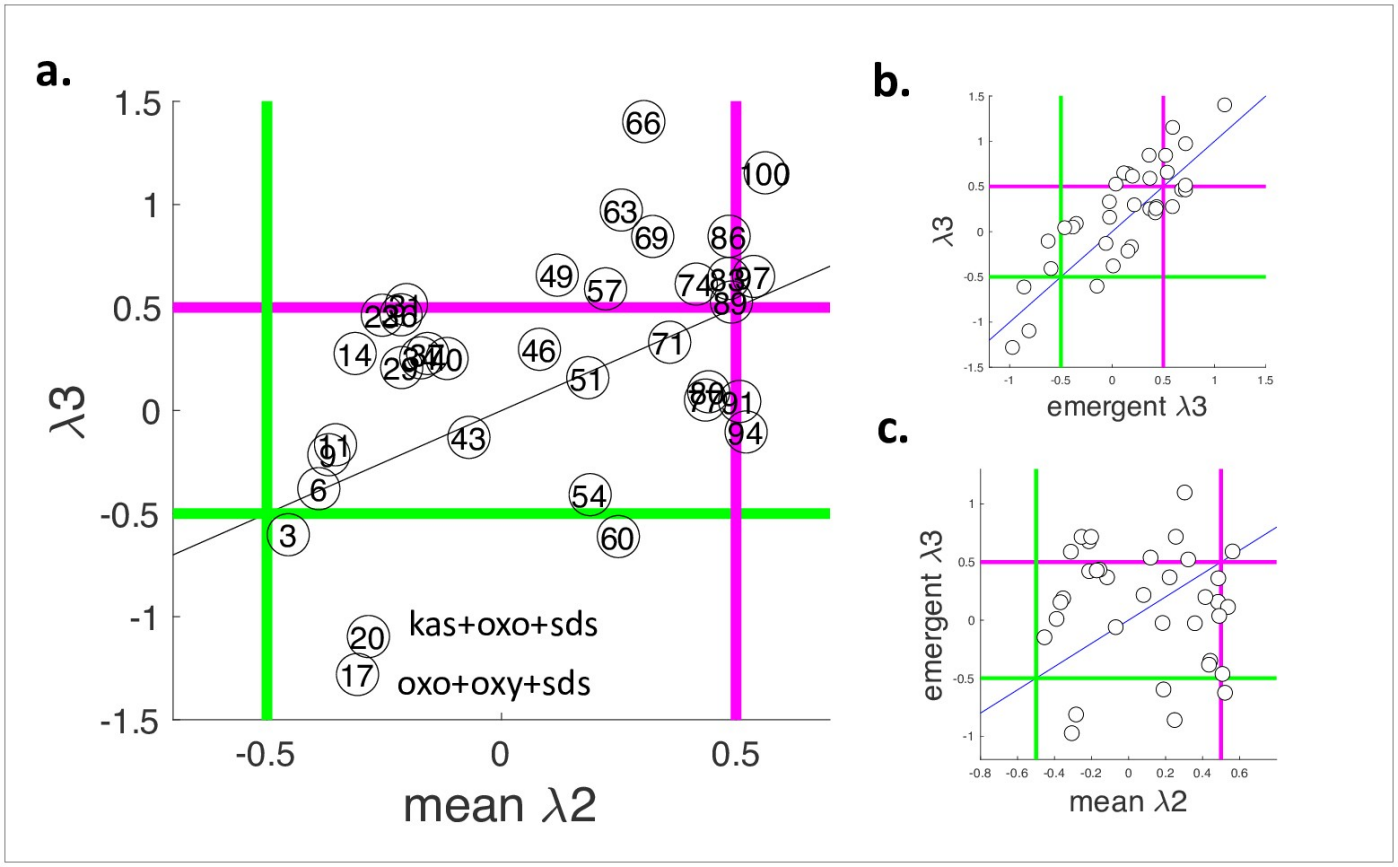
Prediction of three-drug interactions using the average of pairwise interaction scores. (a) Each circle represents a tested three-drug interaction (A+B+C). x-axis shows the mean of pairwise interaction scores (A+B, A+C, B+C) λ2 and y-axis shows the empirically obtained three-drug interaction score λ3. Mean(λ2) and λ3 significantly correlated, indicating that three-drug interactions can be predicted using only pairwise interaction scores (Spearman r = 0.50, P-value = 2.1 x 10^−3^). kas+oxo+SDS and oxo+oxy+SDS were the strongest three-drug synergies. On each three-drug interaction, the percentile among mean(λ2) scores is indicated. kas+oxo+SDS and oxo+oxy+SDS are in the top 20 percentile. Green and magenta lines correspond to synergy (λ < -0.5) and antagonism (λ > 0.5) thresholds. (b) Three-drug interaction scores λ3 and emergent interaction scores (λ3—mean(λ2)) strongly correlate, indicating that emergent interactions contribute to three-drug interactions (Spearman r = 0.75, P-value = 1.7 x 10^−7^). (c) Mean(λ2) and emergent interaction scores had no significant correlation.

Fig 4a shows a scatter plot for predicted scores mean (λ2) and experimental scores (λ3) for all 35 combinations used in the screen. mean(λ2) and λ3 significantly correlated (Spearman r = 0.5, p < 0.01), indicating that pairwise interaction scores provide reliable estimations for three-drug interactions. Therefore, screening only a subset of three-drug combinations guided by pairwise interactions emerges as an efficient platform to discover three-drug synergies. For each three-drug combination depicted in Fig 4a, their percentile in mean(λ2) ranking is indicated. For example, the combination that is predicted to have the lowest λ3 is at ~3 percentile, since there are 35 combinations considered. Of the 35 three-drug combinations, two were found to have the strongest synergies (kas+oxo+SDS) and (oxo+oxy+SDS), and were in the top 20^th^ percentile of predicted synergies. These results show that testing only the top seven combinations predicted by our method would have been sufficient to identify the most synergistic three-drug combinations in the entire three-drug combination space, which corresponds to a five-fold increase in the efficiency of synergy screening.

To test specificity, we used shuffled pairwise interaction data to predict 3-drug interaction scores. In 100,000 simulations, predictions derived from shuffled pairwise data sets showed a correlation equal to or more than real data in 3% of the cases. In addition, we used real pairwise interaction data to predict shuffled 3-drug interaction scores. In 100,000 simulations, predictions showed a correlation equal or more than real data in 1.2% of the cases. The corresponding p-values 0.03 and 0.01 attest to the specificity of our predictions.

The difference between mean (λ2) and λ3 scores has been named as emergent synergy, since this interaction emerges only as a result of the three-drug combination [13, 27]. For each three-drug combination, we computed the emergent λ3 score by = λ3 –mean(λ2). Fig 4b shows that emergent λ3 scores highly correlate with λ3 (Spearman r = 0.75, p < 0.01). Therefore, emergent interactions play an important role for high-order interactions. Emergent interactions do not significantly correlate with mean(λ2). In other words, combinations of synergistic pairs do not result in even more synergistic three-drug interactions. Importantly, the distribution of emergent interactions is skewed towards positive values, therefore three-drug interactions are overall more antagonistic than what is expected from pairwise interactions.

## Discussion

A comprehensive experimental screen for high-order synergies is currently unfeasible, even for relatively small sets of drugs. For example, when 20 drugs are considered, there are 1140 three-drug combinations. Recent studies have shown that pairwise interaction scores provide reliable estimates for three-drug interactions. There are 190 pairwise combinations among 20 drugs. For n drugs, there are (n choose 3) possible three-drug combinations and (n choose 2) possible 2-way combinations. (n choose 3) is (n-2)/3 times larger than (n choose k). For n = 20, there are 6 times more three-drug interactions than pairwise interactions. Therefore, the experimental measurement of only pairwise combinations, to prioritize synergistic three-drug candidates is a strategy for efficiently discovering three-drug synergies. Here, we formalized this approach by (i) measuring all pairwise interactions by checkerboard assay, (ii) measuring all pairwise interactions by diagonal assay, (iii) establishing that diagonal method measures the same phenomenon as the traditional checkerboard assay, (iv) predicting three-drug synergy scores using pairwise diagonal method scores, and (v) testing the three-drug predictions using the diagonal method. Since we have already established the correlation between checkerboard assay and diagonal assay, further iterations of this search strategy may exclude checkerboard assays altogether to discover synergistic three-drug combinations with minimal cost.

A comparison of the checkerboard and diagonal assays strongly argues that the diagonal assay should be the first line of experimentation for synergy screens. Checkerboard assays may then be used to evaluate a combination of interest for validation or discovery of asymmetric interactions. For pairwise interactions, diagonal and checkerboard assays use drug concentration gradients in 1D and 2D, respectively. The experimental setup of a 2D gradient is more difficult than a 1D gradient. For data interpretation, checkerboard assays use isophenotypic contour analysis, which is difficult to perform. Interpretation of diagonal assay is a simple division of observed and expected dose-fractions. Whereas checkerboard assays use more resources than diagonal assays for similar information, diagonal assays are more sensitive than checkerboard assays for equivalent resource cost. In our miniaturized checkerboard assays, we used 4 gradients of each drug and 4x4 = 16 microplate wells per combination. In our diagonal assays, we used 8 gradients of each drug (and combination) and used 8x3 = 24 microplate wells per combination. Despite only 33% increase in experimental cost, the diagonal assay has 2X the resolution of the miniaturized checkerboard assay. This is represented by the stronger correlation among replicates for the diagonal assay (0.79), as compared with the replicate correlation of the mini-checkerboard assay (0.73). For high-order combinations, checkerboard assay quickly becomes prohibitively expensive, necessitating the use of diagonal method. A miniaturized 3D checkercube assay would have 4x4x4 = 64 wells. However, measurement of a three-drug interaction with the diagonal method using an 8-dose gradient only takes 4x8 = 32 wells. Even for three-drug interactions, the diagonal method is both cheaper and more sensitive than mini-checkerboard assays.

The observation that pairwise interaction scores provide reliable estimates for three-drug interactions can be formally expressed by the disentanglement of each factor that contributes to the three-drug interaction. When three drugs A, B, and C are combined, all three pairwise interactions (A+B, A+C, B+C) and one three-drug emergent interaction (A+B+C) occur and the observed interaction is a sum of all these interactions, which can be factorized as: λ3 = mean(λ2) + emergent λ3. Our study is in agreement with previous studies that show that mean(λ2) is not correlated with emergent λ3 [13]. Currently, emergent synergy is computationally unpredictable and experimentally more expensive than measuring the combination’s nominal synergy. Importantly, emergent λ3 is distributed around 0 with a tendency for positive values. Therefore, in the absence of emergent λ3 information, most three-drug combinations will be expected to be slightly more antagonistic than the mean of pairwise interactions.

We importantly note that we have thus far only considered synergy/antagonism of the efficacy of a drug combination. Drugs and their combinations may have side effects such as toxicity, and a combination may have synergistic/antagonistic toxicity. Therefore, a good combination treatment would have synergistic efficacy while not having synergistic toxicity [28, 29, 30, 31]. In a recent study we showed that combinations that have synergistic activity for both desired and undesired phenotypes, cautioning against the side effects of synergistic combinations [32]. A good rule-of-thumb while designing combinations is choosing synergistic combinations with non-overlapping toxicity. This design element is similar to choosing combinations with non-overlapping resistance mechanisms [1, 2, 8]. Since both heuristics are informed only by experimentation on single drugs, their solution does not require experimental screens of large number of combinations. Therefore, the guided screen presented here may be extended by the consideration of non-overlapping toxicity and resistance at minimal cost.

While our study is aimed to describe a general method for finding three-drug synergies for any pathogen or tumor, the results we have obtained for *E*. *amylovora* are also noteworthy. As in numerous previous screens, we have found that antagonism is common, and synergy is very rare [8, 23, 24]. This attests to the need for prudent design of synergistic drug combinations. The only synergies we identified in our pairwise or three-drug screens involved SDS, which we have included for its synergy inducing properties. Combining antibiotics with soap, of course, is not a viable option for systemic infections in humans. However, SDS is recognized as safe for food use by FDA and is used as a control agent for a fungal pathogen to reduce the white rust of spinach [33]. For this reason, use of SDS+antibiotic combinations in the management of plant infections such as fire blight might indeed be practical and as we have seen in our study, may result in synergistic therapeutic benefits to the plant. A next step may include application of combining SDS with other antibiotics in greenhouse and field experiments.

## Materials and methods

### Experimental materials and methods

Copper (II) sulfate (Lot # BCBP2427V), gentamicin sulfate (Lot # SLBK1443V), kasugamycin (Lot # BCBS4283V), oxolinic acid (Lot # BCBH6650V), oxytetracycline hydrochloride (Lot # BCBG9599V), pentamidine isethionate salt (Lot # 117K3731V) and streptomycin sulfate (Lot # 081M13801V) were purchased from Sigma. Sodium dodecyl sulfate (Product No: M82553115) was obtained from Molekula. All drugs were dissolved in water from Millipore MilliQ ultrapure water system and kept at -20°C. All experiments were conducted with *Erwinia amylovora* (Burill) ATCC 49946. Cells were grown in LB at 26°C for 16 hours and diluted to OD600: 0.01. Optical densities of the cells were measured using a plate reader (Tecan Microplate Reader). This microplate reader keeps the temperature at 26°C and provides air flow by shaking the plates in every 15 minutes. MATLAB code was used to obtain the growth curves from the numerical data.

### Determination of the Minimum inhibitory Concentration (MIC)

The MIC of eight drugs against *E*. *amylovora* was determined by using a cell growth assay in 96- well culture plates using LB growth media, with logarithmic, and then linear dilution of drugs. An automated plate reader (Tecan Microplate Reader) was used to quantitatively measure the turbidity in all wells, and the concentration at which there is no increase in turbidity (no cell growth) was identified as the MIC for that drug. The MIC determined by this method corresponds to the concentration at which the drug is bacteriostatic.

### 4 × 4 checkerboard assay

After determining the individual MIC of each drugs, pairwise interactions were evaluated according to the method of “Miniaturized Checkerboard Assays to Measure Antibiotic Interactions” [11]. For this, the concentration of each individual drug was linearly increased on each axis ranging from zero to MIC in a 4x4 matrix. All assays were performed in duplicate. OD600 readings was measured in a TECAN Infinite F200 microplate reader every 15 min at 26°C for 16 hours. The α-score for each 4x4 assay were quantified using the isophenotypic growth contour method described [23]. The results were interpreted as synergy (α < -1) and antagonism (α > 1) thresholds. If the growth contour was linear, concavity or convexity, interactions were classified as additive, synergistic or antagonistic, respectively.

### Diagonal assays

All pairwise interactions were performed according to the method by “Diagonal Method to Measure Synergy Among Any Number of Drugs” for establishing that diagonal method measures the same phenomenon as the traditional checkerboard assay [12]. This method requires two single and one pairwise drug dose-response, in total. For this, concentration of each individual drugs and an equipotent mixture of these drugs (1:1 v/v) were linearly increased in one axis into 96 well plate. *E*. *amylovora* were grown in LB media in the presence of combinations of eight drugs. OD600 measurements using a TECAN microplate reader were done every 15 min at 26°C for 16 hours. Dose-response curves were generated using MATLAB. The expected dose of the two-drug combination was calculated as the intersection of this two-drug-dose-response in the dose combination space with the straight line defined by the IC50 values of each single drug dose-response [34]. The observed dose of the combination is the interpolated IC50 of the two-drug dose-response. λ was calculated using the formula: λ2 = log2 (obs IC50 / exp IC50). The results were interpreted as synergy (λ2 < -0.5) and antagonism (λ2 > 0.5) thresholds.

The synergy scores obtained in pairwise interaction was a guidance in discovery of three-way combinations. For testing the three-drug predictions using diagonal method, the concentration of each drugs and equipotent mixture of these drugs (1:1:1 v/v) were linearly increased in one axis into 96 well plate. Bacteria were treated with the combinations of eight drugs. λ was calculated using the formula: λ3 = log2 (obs IC50 / exp IC50). The results were interpreted as synergy (λ3 < -0.5) and antagonism (λ3 > 0.5) thresholds.

All pairwise interaction experiments (checkerboard and diagonal testing) were done in two biological replicates. The agreement between checkerboard replicates (r = 0.73, p < 0.01) or diagonal replicates (r = 0.83, p < 0.01) are given in Figs 1 or 2, respectively. Our 3-drug predictions were based on diagonal scores, which were significantly but not perfectly correlated. To test predictions for 3-drugs, we calculated the correlation between predictions and empirical interaction scores. Importantly, the correlation between predictions and experiments cannot be higher than the correlation among experiments. Therefore, the highest correlation we may expect from prediction-experiment comparison is 0.83 (correlation among replicates of pairwise diagonal experiments). The correlation of 0.5 between prediction-experiment was able to capture 0.5/0.83*100 = 60% of the predictable correlation despite the underlying noise in the data.
