# Supplementary material for: Guided screen for synergistic three-drug combinations
Source: PLoS One. 2020 Jul 9;15(7):e0235929. doi: 10.1371/journal.pone.0235929 (PMC7347197; doi:10.1371/journal.pone.0235929)
Supplement: Peer review history

# Peer review: Decision Letter 0

**DOI:** 10.1371/journal.pone.0235929.r001

**Authors:** Luis Eduardo M Quintas

21 May 2020

PONE-D-20-08982

Guided screen for synergistic three-drug combinations

PLOS ONE

Dear Dr. Cokol,

Thank you for submitting your manuscript to PLOS ONE. After careful consideration, we feel that it has merit but does not fully meet PLOS ONE’s publication criteria as it currently stands. Therefore, we invite you to submit a revised version of the manuscript that addresses the points raised during the review process. More specifically, reviewer #3 made important comments on the statistical analysis.

We would appreciate receiving your revised manuscript by Jul 05 2020 11:59PM. When you are ready to submit your revision, log on to https://www.editorialmanager.com/pone/ and select the 'Submissions Needing Revision' folder to locate your manuscript file.

If you would like to make changes to your financial disclosure, please include your updated statement in your cover letter.

To enhance the reproducibility of your results, we recommend that if applicable you deposit your laboratory protocols in protocols.io, where a protocol can be assigned its own identifier (DOI) such that it can be cited independently in the future. For instructions see: http://journals.plos.org/plosone/s/submission-guidelines#loc-laboratory-protocols

Please include the following items when submitting your revised manuscript:

- A rebuttal letter that responds to each point raised by the academic editor and reviewer(s). This letter should be uploaded as separate file and labeled 'Response to Reviewers'.
- A marked-up copy of your manuscript that highlights changes made to the original version. This file should be uploaded as separate file and labeled 'Revised Manuscript with Track Changes'.
- An unmarked version of your revised paper without tracked changes. This file should be uploaded as separate file and labeled 'Manuscript'.

Please note while forming your response, if your article is accepted, you may have the opportunity to make the peer review history publicly available. The record will include editor decision letters (with reviews) and your responses to reviewer comments. If eligible, we will contact you to opt in or out.

We look forward to receiving your revised manuscript.

Kind regards,

Luis Eduardo M Quintas, Ph.D.

Academic Editor

PLOS ONE

Journal Requirements:

When submitting your revision, we need you to address these additional requirements.

1. Please ensure that your manuscript meets PLOS ONE's style requirements, including those for file naming. The PLOS ONE style templates can be found at https://journals.plos.org/plosone/s/file?id=wjVg/PLOSOne_formatting_sample_main_body.pdf and https://journals.plos.org/plosone/s/file?id=ba62/PLOSOne_formatting_sample_title_authors_affiliations.pdf

2. Thank you for stating the following in the Financial Disclosure section:

The funders had no role in study design, data collection and analysis, decision to

publish, or preparation of the manuscript. 

We note that one or more of the authors are employed by a commercial company: Axcella Health.

a. Please provide an amended Funding Statement declaring this commercial affiliation, as well as a statement regarding the Role of Funders in your study. If the funding organization did not play a role in the study design, data collection and analysis, decision to publish, or preparation of the manuscript and only provided financial support in the form of authors' salaries and/or research materials, please review your statements relating to the author contributions, and ensure you have specifically and accurately indicated the role(s) that these authors had in your study. You can update author roles in the Author Contributions section of the online submission form.

Please also include the following statement within your amended Funding Statement.

“The funder provided support in the form of salaries for authors [insert relevant initials], but did not have any additional role in the study design, data collection and analysis, decision to publish, or preparation of the manuscript. The specific roles of these authors are articulated in the ‘author contributions’ section.”

If your commercial affiliation did play a role in your study, please state and explain this role within your updated Funding Statement.

b. Please also provide an updated Competing Interests Statement declaring this commercial affiliation along with any other relevant declarations relating to employment, consultancy, patents, products in development, or marketed products, etc. 

Within your Competing Interests Statement, please confirm that this commercial affiliation does not alter your adherence to all PLOS ONE policies on sharing data and materials by including the following statement: "This does not alter our adherence to  PLOS ONE policies on sharing data and materials.” (as detailed online in our guide for authors http://journals.plos.org/plosone/s/competing-interests) . If this adherence statement is not accurate and  there are restrictions on sharing of data and/or materials, please state these. Please note that we cannot proceed with consideration of your article until this information has been declared.

Please include both an updated Funding Statement and Competing Interests Statement in your cover letter. We will change the online submission form on your behalf.

Please know it is PLOS ONE policy for corresponding authors to declare, on behalf of all authors, all potential competing interests for the purposes of transparency. PLOS defines a competing interest as anything that interferes with, or could reasonably be perceived as interfering with, the full and objective presentation, peer review, editorial decision-making, or publication of research or non-research articles submitted to one of the journals. Competing interests can be financial or non-financial, professional, or personal. Competing interests can arise in relationship to an organization or another person. Please follow this link to our website for more details on competing interests: http://journals.plos.org/plosone/s/competing-interests

[Note: HTML markup is below. Please do not edit.]

Reviewers' comments:

Reviewer's Responses to Questions

**Comments to the Author**

1. Is the manuscript technically sound, and do the data support the conclusions?

The manuscript must describe a technically sound piece of scientific research with data that supports the conclusions. Experiments must have been conducted rigorously, with appropriate controls, replication, and sample sizes. The conclusions must be drawn appropriately based on the data presented. 

Reviewer #1: Yes

Reviewer #2: Yes

Reviewer #3: Yes

**********

2. Has the statistical analysis been performed appropriately and rigorously? 

Reviewer #1: Yes

Reviewer #2: Yes

Reviewer #3: No

**********

3. Have the authors made all data underlying the findings in their manuscript fully available?

The PLOS Data policy requires authors to make all data underlying the findings described in their manuscript fully available without restriction, with rare exception (please refer to the Data Availability Statement in the manuscript PDF file). The data should be provided as part of the manuscript or its supporting information, or deposited to a public repository. For example, in addition to summary statistics, the data points behind means, medians and variance measures should be available. If there are restrictions on publicly sharing data—e.g. participant privacy or use of data from a third party—those must be specified.

Reviewer #1: Yes

Reviewer #2: Yes

Reviewer #3: Yes

**********

4. Is the manuscript presented in an intelligible fashion and written in standard English?

PLOS ONE does not copyedit accepted manuscripts, so the language in submitted articles must be clear, correct, and unambiguous. Any typographical or grammatical errors should be corrected at revision, so please note any specific errors here.

Reviewer #1: Yes

Reviewer #2: Yes

Reviewer #3: Yes

**********

5. Review Comments to the Author

Please use the space provided to explain your answers to the questions above. You may also include additional comments for the author, including concerns about dual publication, research ethics, or publication ethics. (Please upload your review as an attachment if it exceeds 20,000 characters)

Reviewer #1: Methods to better predict higher order drug-drug interactions has multiple applications. Cokol and colleagues have developed several of the methods that have made significant advances in this field. Here, the authors look at 3 drug interactions for 7 treatments for the plant pathogen Erwinia. They identify that only the detergent SDS reliably results in synergistic interactions, with many antagonistic interactions, cautioning against a rationale that ‘multi-drug’ therapy is always superior.

Overall, the study is well-performed and ready for publication.

My only recommendation is that the title is too general, and needs to state that this screen was performed in Erwinia.

Babak Javid

Reviewer #2: The article by Cokol-Cakmak and colleagues addresses an important, old, and for a long time unsolved problem in pharmacology, namely how to choose among the astronomically large number of permutations of multiple drugs that could potentially be used in a combination therapy.

In the domain of antimicrobial and anti-cancer chemotherapy, recent discoveries from multiple groups (including from the senior author) have suggested a possible solution. Specifically, it has been observed in each of bacteria, fungi, and cancer that the most substantial drug interactions occur at the 'pairwise' level, whereas 'higher-order' interactions (among 3 or more drugs) are comparatively minor. The implication of this observation is that the combined potency of cocktails of 3 or more drugs may be predictable from measurement of drug pairs. The current manuscript tests this idea and demonstrates how a synthesis of experiments and predictions makes it possible to systematically search a very large number of potential combinations and find the most strongly synergistic many-drug combinations.

The proposed approach consists of

1) experimentally measure pairwise drug interactions

(using a very experimentally efficient 'diagonal' approach)

2) use pairwise data to predict the effects of high-order combinations

3) experimentally test most promising high-order combinations

This approach is novel and eminently sensible, and I think this article describing and demonstrating this approach will be an important contribution to the field. I am grateful that the approach does not rely on prediction alone, but uses prediction to guide experimental measurements to the most promising options. Theory plays a role but the final result is empirical.

With respect to the potential generality and applicability of the observations, two points are worth noting in review. First, the pharmacological principles that guide this approach have been supported in many different diseases and model systems, and therefore I am satisfied that this proposal on how to practically apply these principles is one that has general interest beyond the particular model system being studied. (The application here to a plant pathogen is outside of my expertise but looks interesting). Second, the use of 'many-drug' combinations is routine in many areas of infectious disease and cancer medicine, so this topic certainly has medical relevance. Any use of novel combinations in humans of course requires phase 1 trials of safety, and combination design for human use should involve considerations of overlapping or non-overlapping toxicities. I think if one were to apply the approach described here to therapies for human use, the approach could take this into account, by narrowing the search space to only one agent per mode of dose-limiting toxicity (i.e. only one agent with dose-limiting hepatotoxicity, only one with dose-limiting cardiotoxicity, etc). So while toxicity is an important consideration in developing human combination therapies it does not override the practicality of the approach here, because these aspects could be considered simultaneously in prediction-guided experiments. Of course this is not relevant to Erwinia amylovora, and I leave it to the authors to decide if they should like to mention these considerations in the discussion.

The most substantial advance of the work is describing and demonstrating the approach to searching for synergistic multidrug combinations. For me the experimental findings in Erwinia amylovora are of secondary significance. The drug interactions observed in this system are of small magnitude making the overall effect of synergy minor. The measurements of pairwise interactions show substantial variation among replicates, although not worse than is typical of high-throughput screens. Although experimental noise in pairwise interactions will propagate into noisy predictions, this concern is overridden by the study ultimately conducting experimental measurements of the high-order combinations of interest. Overall, the strategy described here is interesting, theoretically rigorous, and useful. I expect this article will have general relevance to many efforts to find superior combination therapies for the treatment of pathogens or other diseases.

Reviewer #3: This is an interesting article that may have substantial impact on design of drug combination screens particularly those involving higher order combinations. The article is well written and overall has sufficient clarity. The experiment designs are interesting and does not suffer from any major deficiency to the best of my understanding. There is however need for increased statistical rigor and clarification in certain parts of the paper.

1. The authors claim that their results are “in agreement with numerous studies.” With no justification and in same instances not even a citation. The authors should clarify and detail the nature of the agreement, cite appropriate papers and also justify such agreements. There is also a need for discussion on what such agreements would imply.

2. The data is fairly interesting yet I have not seen in a detailed analysis of biological or technical replicates. The authors need to provide a detailed account of the fit between replicates particularly in the paired combinations and compare the variation/noise in the underlying data to the accuracy of predictions.

3. The authors predicted higher order drug-drug interactions based on the average of paired drug interactions. They explained their strategy as “For all three-drug combinations of these 8 compounds, we generated predictions by using the arithmetic mean of interaction scores for all 3 pairwise combinations among 3 drugs. For combination A+B+C, the mean (AB, AC, BC) provides the expectation for A+B+C if there is no additional synergy/antagonism associated with the three-drug combination of these drugs”. This is an interesting yet extremely simple method. This approach needs better explanation/justification. How did the authors chose this simple approach, what are the examples in the literature, how does their method compare to others? Even If they do not provide a detailed benchmark, a good discussion and scientific justification would strengthen the paper.

4. The prediction power seems to be sufficiently strong based on the analysis in the manuscript. Yet we do not know about the specificity f the predictions. The authors should take a bootstrapping approach to test the predictive power of shuffled data. The predictions from shuffled data will generate a null model of predictions and statistical comparison of their actual predictions to this null model can provide better insight on the value of the predictions.

**********

6. PLOS authors have the option to publish the peer review history of their article (what does this mean?). If published, this will include your full peer review and any attached files.

If you choose “no”, your identity will remain anonymous but your review may still be made public.

**Do you want your identity to be public for this peer review?** For information about this choice, including consent withdrawal, please see our Privacy Policy.

Reviewer #1: No

Reviewer #2: No

Reviewer #3: No

[NOTE: If reviewer comments were submitted as an attachment file, they will be attached to this email and accessible via the submission site. Please log into your account, locate the manuscript record, and check for the action link "View Attachments". If this link does not appear, there are no attachment files to be viewed.]

While revising your submission, please upload your figure files to the Preflight Analysis and Conversion Engine (PACE) digital diagnostic tool, https://pacev2.apexcovantage.com/. PACE helps ensure that figures meet PLOS requirements. To use PACE, you must first register as a user. Registration is free. Then, login and navigate to the UPLOAD tab, where you will find detailed instructions on how to use the tool. If you encounter any issues or have any questions when using PACE, please email us at figures@plos.org. Please note that Supporting Information files do not need this step.

# Author comment: Author response to Decision Letter 0

**DOI:** 10.1371/journal.pone.0235929.r002

19 Jun 2020

Reviewer #1: Methods to better predict higher order drug-drug interactions has multiple applications. Cokol and colleagues have developed several of the methods that have made significant advances in this field. Here, the authors look at 3 drug interactions for 7 treatments for the plant pathogen Erwinia. They identify that only the detergent SDS reliably results in synergistic interactions, with many antagonistic interactions, cautioning against a rationale that ‘multi-drug’ therapy is always superior.

Overall, the study is well-performed and ready for publication.

My only recommendation is that the title is too general, and needs to state that this screen was performed in Erwinia.

Babak Javid

We thank the reviewer for his encouraging comments. As all the reviewers noted, the method outlined in our study has applications in multiple fields ranging from antimicrobials, cancer and metabolic diseases. In reviewer 2’s words: “…this proposal on how to practically apply these principles is one that has general interest beyond the particular model system being studied”. We fear that making our title too specific may limit our audience. As we stated in our abstract, the screen is made in Erwinia amylovora, which is just a model for establishing a more general method. For these reasons, we would like to respectfully keep our original title and leave it to the editor’s discretion if a more specific title such as “Guided screen for three-drug combinations in Erwinia amylovora, the causative agent of fire blight” should be used.

Reviewer #2: The article by Cokol-Cakmak and colleagues addresses an important, old, and for a long time unsolved problem in pharmacology, namely how to choose among the astronomically large number of permutations of multiple drugs that could potentially be used in a combination therapy.

In the domain of antimicrobial and anti-cancer chemotherapy, recent discoveries from multiple groups (including from the senior author) have suggested a possible solution. Specifically, it has been observed in each of bacteria, fungi, and cancer that the most substantial drug interactions occur at the 'pairwise' level, whereas 'higher-order' interactions (among 3 or more drugs) are comparatively minor. The implication of this observation is that the combined potency of cocktails of 3 or more drugs may be predictable from measurement of drug pairs. The current manuscript tests this idea and demonstrates how a synthesis of experiments and predictions makes it possible to systematically search a very large number of potential combinations and find the most strongly synergistic many-drug combinations.

The proposed approach consists of

1) experimentally measure pairwise drug interactions

(using a very experimentally efficient 'diagonal' approach)

2) use pairwise data to predict the effects of high-order combinations

3) experimentally test most promising high-order combinations

This approach is novel and eminently sensible, and I think this article describing and demonstrating this approach will be an important contribution to the field. I am grateful that the approach does not rely on prediction alone, but uses prediction to guide experimental measurements to the most promising options. Theory plays a role but the final result is empirical.

With respect to the potential generality and applicability of the observations, two points are worth noting in review. First, the pharmacological principles that guide this approach have been supported in many different diseases and model systems, and therefore I am satisfied that this proposal on how to practically apply these principles is one that has general interest beyond the particular model system being studied. (The application here to a plant pathogen is outside of my expertise but looks interesting). Second, the use of 'many-drug' combinations is routine in many areas of infectious disease and cancer medicine, so this topic certainly has medical relevance. Any use of novel combinations in humans of course requires phase 1 trials of safety, and combination design for human use should involve considerations of overlapping or non-overlapping toxicities. I think if one were to apply the approach described here to therapies for human use, the approach could take this into account, by narrowing the search space to only one agent per mode of dose-limiting toxicity (i.e. only one agent with dose-limiting hepatotoxicity, only one with dose-limiting cardiotoxicity, etc). So while toxicity is an important consideration in developing human combination therapies it does not override the practicality of the approach here, because these aspects could be considered simultaneously in prediction-guided experiments. Of course this is not relevant to Erwinia amylovora, and I leave it to the authors to decide if they should like to mention these considerations in the discussion.

The most substantial advance of the work is describing and demonstrating the approach to searching for synergistic multidrug combinations. For me the experimental findings in Erwinia amylovora are of secondary significance. The drug interactions observed in this system are of small magnitude making the overall effect of synergy minor. The measurements of pairwise interactions show substantial variation among replicates, although not worse than is typical of high-throughput screens. Although experimental noise in pairwise interactions will propagate into noisy predictions, this concern is overridden by the study ultimately conducting experimental measurements of the high-order combinations of interest. Overall, the strategy described here is interesting, theoretically rigorous, and useful. I expect this article will have general relevance to many efforts to find superior combination therapies for the treatment of pathogens or other diseases.

We thank the reviewer for their kind words and their appreciation of our study in the general context of synergy search. We revised our discussion to include the following sentences on overlapping toxicity while designing combinations in humans and a citation to a recent article on the effect of drug interactions on therapeutic selectivity. 

“We importantly note that we have thus far only considered synergy/antagonism of the efficacy of a drug combination. Drugs and their combinations may have side effects such as toxicity, and a combination may have synergistic/antagonistic toxicity. Therefore, a good combination treatment would have synergistic efficacy while not having synergistic toxicity. In a recent study we showed that combinations that have synergistic activity for both desired and undesired phenotypes, cautioning against the side effects of synergistic combinations. A good rule-of-thumb while designing combinations is choosing synergistic combinations with non-overlapping toxicity. This design element is similar to choosing combinations with non-overlapping resistance mechanisms. Since both heuristics are informed only by experimentation on single drugs, their solution does not require experimental screens of large number of combinations. Therefore, the guided screen presented here may be extended by the consideration of non-overlapping toxicity and resistance at minimal cost.”

Reviewer #3: This is an interesting article that may have substantial impact on design of drug combination screens particularly those involving higher order combinations. The article is well written and overall has sufficient clarity. The experiment designs are interesting and does not suffer from any major deficiency to the best of my understanding. There is however need for increased statistical rigor and clarification in certain parts of the paper.

1. The authors claim that their results are “in agreement with numerous studies.” With no justification and in same instances not even a citation. The authors should clarify and detail the nature of the agreement, cite appropriate papers and also justify such agreements. There is also a need for discussion on what such agreements would imply.

We apologize for this omission and thank the reviewer for pointing it out. In two instances in the text, we have added citations to support that antagonism is common and synergy is rare.

2. The data is fairly interesting yet I have not seen in a detailed analysis of biological or technical replicates. The authors need to provide a detailed account of the fit between replicates particularly in the paired combinations and compare the variation/noise in the underlying data to the accuracy of predictions.

We thank the reviewer for attention to replicates and bringing attention to the relationship between input data noise and prediction accuracy. In the revised version, we highlighted our detailed analysis of replicates and explicitly compared the variation in replicates with prediction accuracy. We added the following sentences in the methods section. 

“All pairwise interaction experiments (checkerboard and diagonal testing) were done in two biological replicates. The agreement between checkerboard replicates (r = 0.73, p < 0.01) or diagonal replicates (r = 0.83, p < 0.01) are given in Figure 1 or Figure 2, respectively. Our 3-drug predictions were based on diagonal scores, which were significantly but not perfectly correlated. To test predictions for 3-drugs, we calculated the correlation between predictions and empirical interaction scores. Importantly, the correlation between predictions and experiments cannot be higher than the correlation among experiments. Therefore, the highest correlation we may expect from prediction-experiment comparison is 0.83 (correlation among replicates of pairwise diagonal experiments). The correlation of 0.5 between prediction-experiment was able to capture 0.5/0.83*100 = 60% of the predictable correlation despite the underlying noise in the data.”

3. The authors predicted higher order drug-drug interactions based on the average of paired drug interactions. They explained their strategy as “For all three-drug combinations of these 8 compounds, we generated predictions by using the arithmetic mean of interaction scores for all 3 pairwise combinations among 3 drugs. For combination A+B+C, the mean (AB, AC, BC) provides the expectation for A+B+C if there is no additional synergy/antagonism associated with the three-drug combination of these drugs”. This is an interesting yet extremely simple method. This approach needs better explanation/justification. How did the authors chose this simple approach, what are the examples in the literature, how does their method compare to others? Even If they do not provide a detailed benchmark, a good discussion and scientific justification would strengthen the paper.

We thank the reviewer for the opportunity to clarify this point. It has been previously published that the mean of pairwise scores provide reliable estimates for a 3-drug interaction score. This is explained by the factorization of a high-order interactions. In the introduction of the revised submission, we included one additional paragraph to describe the rationale behind our prediction method. We referred to this paragraph in the results section when the method is applied.

Additional paragraph in introduction:

“When three drugs A, B, and C are combined, the observed (nominal) interaction has four components: Three pairwise interactions (A+B, A+C, B+C) and the interaction resulting specifically from the combination of all three components (A+B+C). This specific interaction resulting only from the high-order combination has been referred to as “emergent interaction.” These four components can be expressed as: nominal interaction = emergent interaction + mean(three pairwise interactions). Moreover, multiple studies have shown that emergent interactions are rare. Therefore, simple mean of pairwise interactions may provide a reliable estimate for nominal three-drug interaction scores.”

Edited results:

“For all three-drug combinations of these 8 compounds, we generated predictions by using the arithmetic mean of interaction scores for all 3 pairwise combinations among 3 drugs, as introduced above. For combination A+B+C, the mean (AB, AC, BC) provides the expectation for A+B+C if there is no additional synergy/antagonism associated with the three-drug combination of these drugs”.

4. The prediction power seems to be sufficiently strong based on the analysis in the manuscript. Yet we do not know about the specificity f the predictions. The authors should take a bootstrapping approach to test the predictive power of shuffled data. The predictions from shuffled data will generate a null model of predictions and statistical comparison of their actual predictions to this null model can provide better insight on the value of the predictions.

We thank the reviewer for this suggestion. In the revised submission, we include an analysis using shuffled data to test specificity, adding the following sentences to the results section: 

“To test specificity, we used shuffled pairwise interaction data to predict 3-drug interaction scores. In 100,000 simulations, predictions derived from shuffled pairwise data sets showed a correlation equal to or more than real data in 3% of the cases. In addition, we used real pairwise interaction data to predict shuffled 3-drug interaction scores. In 100,000 simulations, predictions showed a correlation equal or more than real data in 1.2% of the cases. The corresponding p-values 0.03 and 0.01 attest to the specificity of our predictions.”

Attachment Submitted filename: ResponseToReviewers_061520.docx Click here for additional data file.

# Peer review: Decision Letter 1

**DOI:** 10.1371/journal.pone.0235929.r003

**Authors:** Luis Eduardo M Quintas

25 Jun 2020

Guided screen for synergistic three-drug combinations

PONE-D-20-08982R1

Dear Dr. Cokol,

We’re pleased to inform you that your manuscript has been judged scientifically suitable for publication and will be formally accepted for publication once it meets all outstanding technical requirements.

Within one week, you’ll receive an e-mail detailing the required amendments. When these have been addressed, you’ll receive a formal acceptance letter and your manuscript will be scheduled for publication.

An invoice for payment will follow shortly after the formal acceptance. To ensure an efficient process, please log into Editorial Manager at http://www.editorialmanager.com/pone/, click the 'Update My Information' link at the top of the page, and double check that your user information is up-to-date. If you have any billing related questions, please contact our Author Billing department directly at authorbilling@plos.org.

If your institution or institutions have a press office, please notify them about your upcoming paper to help maximize its impact. If they’ll be preparing press materials, please inform our press team as soon as possible -- no later than 48 hours after receiving the formal acceptance. Your manuscript will remain under strict press embargo until 2 pm Eastern Time on the date of publication. For more information, please contact onepress@plos.org.

Kind regards,

Luis Eduardo M Quintas, Ph.D.

Academic Editor

PLOS ONE

Additional Editor Comments (optional):

Reviewers' comments:

Reviewer's Responses to Questions

**Comments to the Author**

1. If the authors have adequately addressed your comments raised in a previous round of review and you feel that this manuscript is now acceptable for publication, you may indicate that here to bypass the “Comments to the Author” section, enter your conflict of interest statement in the “Confidential to Editor” section, and submit your "Accept" recommendation.

Reviewer #1: All comments have been addressed

Reviewer #3: All comments have been addressed

**********

2. Is the manuscript technically sound, and do the data support the conclusions?

The manuscript must describe a technically sound piece of scientific research with data that supports the conclusions. Experiments must have been conducted rigorously, with appropriate controls, replication, and sample sizes. The conclusions must be drawn appropriately based on the data presented. 

Reviewer #1: Yes

Reviewer #3: Yes

**********

3. Has the statistical analysis been performed appropriately and rigorously? 

Reviewer #1: Yes

Reviewer #3: Yes

**********

4. Have the authors made all data underlying the findings in their manuscript fully available?

The PLOS Data policy requires authors to make all data underlying the findings described in their manuscript fully available without restriction, with rare exception (please refer to the Data Availability Statement in the manuscript PDF file). The data should be provided as part of the manuscript or its supporting information, or deposited to a public repository. For example, in addition to summary statistics, the data points behind means, medians and variance measures should be available. If there are restrictions on publicly sharing data—e.g. participant privacy or use of data from a third party—those must be specified.

Reviewer #1: Yes

Reviewer #3: Yes

**********

5. Is the manuscript presented in an intelligible fashion and written in standard English?

PLOS ONE does not copyedit accepted manuscripts, so the language in submitted articles must be clear, correct, and unambiguous. Any typographical or grammatical errors should be corrected at revision, so please note any specific errors here.

Reviewer #1: Yes

Reviewer #3: Yes

**********

6. Review Comments to the Author

Please use the space provided to explain your answers to the questions above. You may also include additional comments for the author, including concerns about dual publication, research ethics, or publication ethics. (Please upload your review as an attachment if it exceeds 20,000 characters)

Reviewer #1: Response to the reviewers' comments acceptable and I think the manuscript is now suitable for publication.

Reviewer #3: Thank you for addressing all the points. I think the article is now ready for publication.

The article is now statistically more rigorous.

**********

7. PLOS authors have the option to publish the peer review history of their article (what does this mean?). If published, this will include your full peer review and any attached files.

If you choose “no”, your identity will remain anonymous but your review may still be made public.

**Do you want your identity to be public for this peer review?** For information about this choice, including consent withdrawal, please see our Privacy Policy.

Reviewer #1: No

Reviewer #3: No

# Editor report: Acceptance letter

**DOI:** 10.1371/journal.pone.0235929.r004

**Authors:** Luis Eduardo M Quintas

30 Jun 2020

PONE-D-20-08982R1 

Guided screen for synergistic three-drug combinations 

Dear Dr. Cokol:

I'm pleased to inform you that your manuscript has been deemed suitable for publication in PLOS ONE. Congratulations! Your manuscript is now with our production department. 

If your institution or institutions have a press office, please let them know about your upcoming paper now to help maximize its impact. If they'll be preparing press materials, please inform our press team within the next 48 hours. Your manuscript will remain under strict press embargo until 2 pm Eastern Time on the date of publication. For more information please contact onepress@plos.org.

If we can help with anything else, please email us at plosone@plos.org. 

Thank you for submitting your work to PLOS ONE and supporting open access. 

Kind regards, 

PLOS ONE Editorial Office Staff

on behalf of

Dr. Luis Eduardo M Quintas 

Academic Editor

PLOS ONE
